# Mechanistic Insights into β-Lactamase-Catalysed Carbapenem Degradation Through Product Characterisation

**DOI:** 10.1038/s41598-019-49264-0

**Published:** 2019-09-20

**Authors:** Christopher T. Lohans, Emily I. Freeman, Emma van Groesen, Catherine L. Tooke, Philip Hinchliffe, James Spencer, Jürgen Brem, Christopher J. Schofield

**Affiliations:** 1 Department of Chemistry, University of Oxford, Oxford, OX1 3TA United Kingdom; 2 Department of Biomedical and Molecular Sciences, Queen’s University, Kingston, ON K7L 3N6 Canada; 3 School of Cellular and Molecular Medicine, University of Bristol, Bristol, BS8 1TD United Kingdom

**Keywords:** Hydrolases, Enzyme mechanisms, Antibiotics, Antimicrobial resistance

## Abstract

β-Lactamases are a major threat to the clinical use of carbapenems, which are often antibiotics of last resort. Despite this, the reaction outcomes and mechanisms by which β-lactamases degrade carbapenems are still not fully understood. The carbapenem bicyclic core consists of a β-lactam ring fused to a pyrroline ring. Following β-lactamase-mediated opening of the β-lactam, the pyrroline may interconvert between an enamine (2-pyrroline) form and two epimeric imine (1-pyrroline) forms; previous crystallographic and spectroscopic studies have reported all three of these forms in the contexts of hydrolysis by different β-lactamases. As we show by NMR spectroscopy, the serine β-lactamases (KPC-2, SFC-1, CMY-10, OXA-23, and OXA-48) and metallo-β-lactamases (NDM-1, VIM-1, BcII, CphA, and L1) tested *all* degrade carbapenems to preferentially give the Δ^2^ (enamine) and/or (*R*)-Δ^1^ (imine) products. Rapid non-enzymatic tautomerisation of the Δ^2^ product to the (*R*)-Δ^1^ product prevents assignment of the nascent enzymatic product by NMR. The observed stereoselectivity implies that carbapenemases control the form of their pyrroline ring intermediate(s)/product(s), thereby preventing pyrroline tautomerisation from inhibiting catalysis.

## Introduction

**Figure 1:**
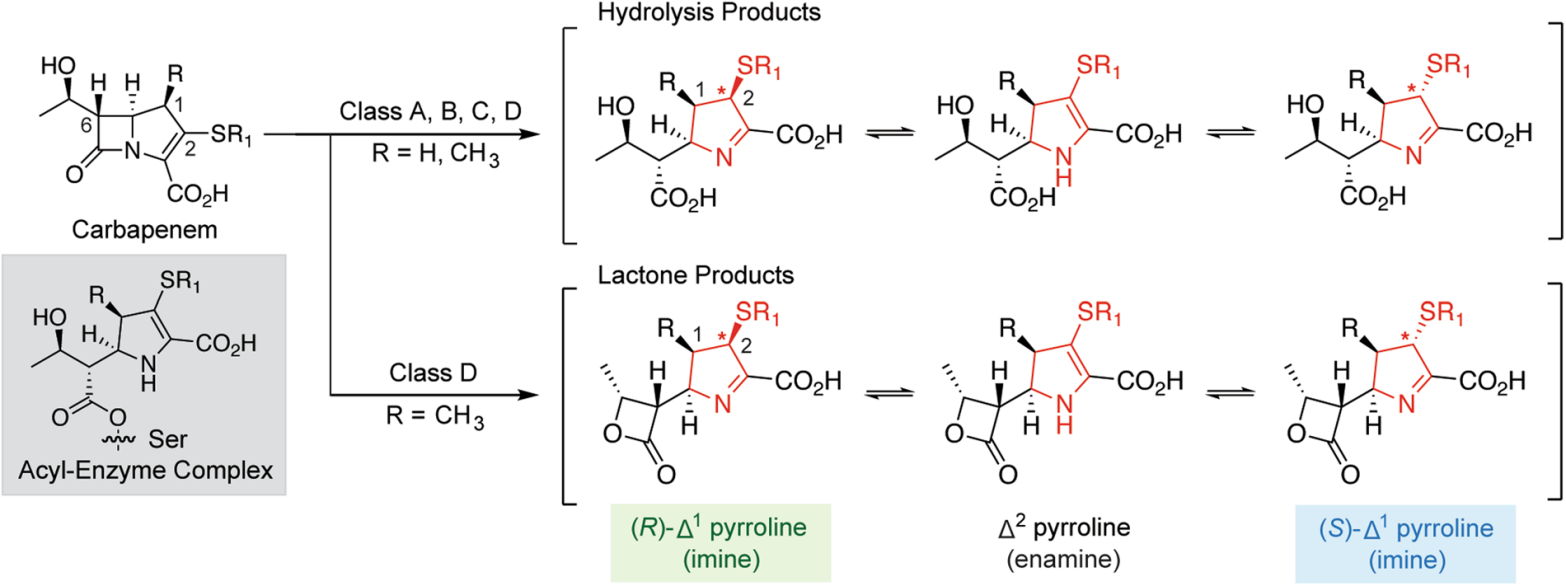
Major products formed following β-lactamase-catalysed carbapenem degradation. The structures of the different tautomeric and epimeric forms of the carbapenem-derived pyrroline ring are shown in red. The stereochemical assignments in brackets refer to the C-2 configuration, indicated with asterisks. The structure of the initial acyl-enzyme complex formed following the nucleophilic attack of serine (in serine β-lactamases or penicillin-binding proteins) with the carbapenem β-lactam ring is shown (shaded in grey).

β-Lactams, including the penicillins, cephalosporins, and carbapenems, are the most widely used antibiotic class^1^. They act by forming covalent complexes with bacterial transpeptidases (penicillin-binding proteins, PBPs), thereby inhibiting cell wall biosynthesis. Of the different β-lactam subclasses, the carbapenems are of particular clinical importance, often being considered to be antibiotics of last resort. Carbapenems have a bicyclic core, consisting of fused β-lactam and pyrroline rings (Fig. 1, Supplementary Fig. S1). In all clinically used carbapenems, this bicyclic core is functionalised with C-2 thioether and C-6 hydroxyethyl sidechains. Although carbapenems with a 1β-hydrogen on the pyrroline ring (e.g., imipenem; R=H in Fig. 1) are susceptible to degradation by human dehydropeptidase^2^, substitution with a 1β-methyl group (e.g., meropenem, ertapenem; R=CH_3_ in Fig. 1) slows this hydrolysis.

Bacterial resistance to β-lactam antibiotics occurs most importantly through the production of β-lactamases^3^. There are two mechanistically distinct groups of β-lactamases: the nucleophilic serine β-lactamases (SBLs) and the metallo-β-lactamases (MBLs). The SBLs are further divided into Ambler classes A, C, and D according to sequence homology and mechanism, while the MBLs are class B^4^. SBLs degrade β-lactam antibiotics via a two-step process. First, the nucleophilic serine residue attacks the β-lactam ring, forming an ester-linked acyl-enzyme complex (Fig. 1). The covalent adduct is then hydrolysed, or, as occurs with class D SBLs and some carbapenems, it may cyclise to give a β-lactone isomeric with the carbapenem substrate (Fig. 1)^5^. The structurally distinct MBLs, however, employ a nucleophilic water molecule, activated by one or two zinc ions, which directly reacts with the β-lactam ring. Although carbapenems were previously thought to be substantially resistant to β-lactamases, the global emergence of enzymes with carbapenemase activity is a major clinical concern^6^.

In the products derived from carbapenem degradation (i.e., hydrolysis and β-lactone products; Fig. 1), the carbapenem pyrroline ring exists in an equilibrium between an enamine (2-pyrroline) form (Δ^2^) and two epimeric imine (1-pyrroline) forms [(*R*)-Δ^1^ and (*S*)-Δ^1^; Fig. 1]^2,5^. This equilibrium has potential to extend to the acyl-enzyme complexes derived from carbapenems and SBLs, where all three forms of the pyrroline ring have been reported in crystallographic analyses with different enzymes^7–11^. Interconversion between the different pyrroline forms appears to be time-dependent, and may occur slowly in the enzyme active site; crystallographic studies of the acyl-enzyme complex derived from the SBL BlaC with the carbapenem ertapenem suggest that the pyrroline ring is initially present in the Δ^2^ enamine form, and tautomerises to the (*S*)-Δ^1^ imine form over time^7^.

In the acyl-enzyme complexes derived from carbapenems and SBLs, tautomerisation of the pyrroline ring from the Δ^2^ form to the Δ^1^ form has been proposed to inhibit hydrolysis^12–14^. Kinetic and spectroscopic studies of the class A SBL RTEM with carbapenems led to the proposal that partitioning between the Δ^2^ and Δ^1^ pyrroline tautomers impacts on the relative hydrolysis rates of the acyl-enzyme complexes^12,13^. Raman studies of the class A SBL SHV-1 with the carbapenem meropenem suggest that the acyl-enzyme complex is susceptible to hydrolysis if the pyrroline ring is in the Δ^2^ form, while conversion to the Δ^1^ form hinders hydrolysis^14^. The apparent stabilisation of the acyl-enzyme complex by formation of the Δ^1^ pyrroline tautomer is proposed to result from the displacement of the substrate carbonyl from the oxyanion hole^14^, or through the disruption of the ‘hydrolytic’ water molecule responsible for deacylation^7^.

The structure of the carbapenem-derived pyrroline ring also has implications for MBL catalysis and inhibition. Following the nucleophilic attack of water onto the β-lactam ring and cleavage of the carbon-nitrogen bond, the resulting anionic species is thought to bind to the MBL active site zinc through its negatively-charged (Δ^2^) pyrroline nitrogen^15^. Stereoselective protonation at C-2 is then proposed to occur, followed by dissociation of the hydrolysed carbapenem from the active site^16,17^. To promote such a mechanism, the active sites of MBLs may be structured such that C-2 is solvent accessible, as proposed for the monozinc MBL Sfh-I^18^.

Although there have been excellent mechanistic studies on the role of the pyrroline ring in carbapenem degradation by SBLs^12,14,19^, most of the enzymes investigated are not efficient carbapenemases. Thus, it was of interest to consider how the structure of the pyrroline ring may relate to carbapenem degradation by SBLs with carbapenemase activity, In particular, we wondered whether carbapenemases are able to degrade both Δ^2^ and Δ^1^ forms of the acyl-enzyme complex (the latter of which has been proposed to be resistant to SBL-catalysed hydrolysis), or if these enzymes may control the tautomeric state of the pyrroline ring such that it remains in the hydrolytically susceptible Δ^2^ form.

## Results

**Figure 2:**
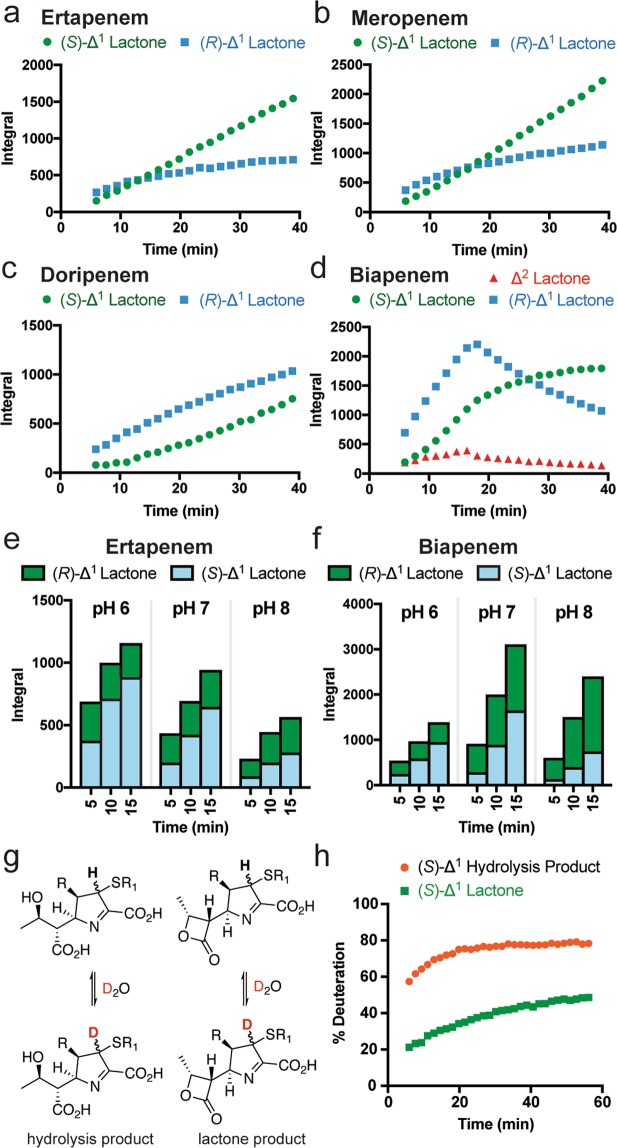
Stereoselectivity of lactone formation, and non-enzymatic interconversion of carbapenem-derived products. NMR time courses showing the observed levels of the (*R*)-Δ^1^, (*S*)-Δ^1^, and Δ^2^ carbapenem-derived lactones produced by OXA-48 (5 µM) with 1 mM (**a**) ertapenem^5^, (**b**) meropenem, (**c**) doripenem, and (**d**) biapenem. Integrals correspond to the methyl group of the C-6 hydroxyethyl side chain. NMR time courses showing the impact of pH on the levels of the (*R*)-Δ^1^ and (*S*)-Δ^1^ carbapenem-derived lactones following addition of OXA-48 (5 µM) to 1 mM (**e**) ertapenem or (**f**) biapenem. Integrals correspond to the methyl group of the C-6 hydroxyethyl side chain. (**g**) Scheme showing exchange of the C-2 hydrogen of carbapenem-derived products in partially deuterated solvent. (**h**) NMR time course showing the extent of deuterium incorporation at C-2 for the meropenem-derived hydrolysis and lactone products dissolved in 50 mM sodium phosphate, pH 7.5, 80% D_2_O, 20% H_2_O. These data indicate that non-enzymatic epimerisation occurs more quickly for carbapenem-derived hydrolysis products than it does for the lactone products.

During our previous NMR spectroscopic studies on β-lactone formation by class D SBLs, we observed the apparent preferential formation of the (*R*)-Δ^1^ lactone product from ertapenem with OXA-48 at early time points (Fig. 2a)^5^. However, at later time points, the products equilibrated to give a ~5:1 mixture of the (*S*)-Δ^1^ and (*R*)-Δ^1^ lactone epimers. We attributed this to non-enzymatic interconversion, favouring the (*S*)-Δ^1^ lactone epimer due to its predicted greater thermodynamic stability (as a consequence of the *trans* orientation of the 1β-methyl group and the C-2 thioether substituent) (Fig. 1). However, preferential formation of the (*R*)-Δ^1^ hydrolysis product was not observed, and an excess of the (*S*)-Δ^1^ hydrolysis product was seen in all measurements^5^.

β-Lactone products were also formed from the carbapenems meropenem, doripenem, and biapenem with OXA-48^5^. To investigate the stereoselectivity of lactone formation from these carbapenems, we used nuclear Overhauser effect spectroscopy (NOESY) NMR experiments to assign the configurations of the lactone products (Supplementary Figs S2–S4; Supplementary Tables S1–S3). Following these assignments, we acquired NMR time courses for meropenem, doripenem, and biapenem showing the lactone products formed by OXA-48 (Fig. 2b–d). While the time course for meropenem and OXA-48 resembled that of ertapenem, the (*R*)-Δ^1^ lactone products derived from biapenem and doripenem clearly predominated over the (*S*)-Δ^1^ lactone products at early time points (Fig. 2). Similar trends were observed for the class D SBL OXA-23 with these carbapenems (Supplementary Fig. S5). In the biapenem OXA-48 product mixture, a third set of signals consistent with a carbapenem-derived lactone was observed by NMR. Further analyses suggested that these signals correspond to the Δ^2^ enamine form of the biapenem-derived lactone (Fig. 2d, Supplementary Table S3). Although related signals were observed for the products of other carbapenems with OXA-48, they were present at far lower levels than those observed for biapenem, precluding further characterisation.

To further investigate the apparently preferred formation of the (*R*)-Δ^1^ lactone product for all studied enzymes, we examined the impact of pH on the product profile of OXA-48 with ertapenem and biapenem (Fig. 2e,f). At higher pH, relatively high levels of the (*R*)-Δ^1^ lactone were observed at early time points, suggesting that interconversion between the (*R*)- and (*S*)- forms occurs more slowly in basic conditions. Sample pH was also observed to impact on the relative levels of hydrolysis and lactone formation; while the levels of lactone formation and hydrolysis were similar at higher pH, a preference for lactone formation was observed at lower pH (Supplementary Fig. S6).

The (*S*)-Δ^1^ form of the ertapenem hydrolysis product predominated in all measurements during an NMR time course with OXA-48^5^. For comparison, we first assigned the chemical shifts of the hydrolysis products derived from meropenem, biapenem, and doripenem (Supplementary Tables S4–S6). In addition to the (*S*)-Δ^1^ and (*R*)-Δ^1^ hydrolysis products, NMR signals consistent with low levels of the Δ^2^ enamine hydrolysis products were observed in these product mixtures (Supplementary Fig. S7). As observed with ertapenem, the (*S*)-Δ^1^ hydrolysis products derived from these carbapenems with OXA-48 predominated at all time points (Supplementary Fig. S8).

As the stereoselectivity of hydrolysis may be anticipated to resemble the stereoselectivity of lactone formation, it was initially unclear why preferential formation of the (*R*)-Δ^1^ hydrolysis product was not observed at early time points. However, this discrepancy could be resolved if the carbapenem-derived hydrolysis products were to undergo non-enzymatic epimerisation more rapidly than the lactone products. The C-2 proton of both lactone and hydrolysis products exchanges with solvent (Fig. 2g)^5^, allowing for the use of NMR to monitor the relative rates of epimerisation in the hydrolysis and lactone products.

The lactone and hydrolysis products derived from meropenem were purified, prepared in partially deuterated buffer, and exchange of the C-2 hydrogen with deuterium was monitored by NMR. While rapid deuterium incorporation was observed for the meropenem-derived hydrolysis product(s), it occurred more slowly for the meropenem-derived lactone(s) (Fig. 2h). These observations indicate that the meropenem hydrolysis products indeed epimerise more rapidly than the corresponding lactone products; this difference may reflect activation of the pyrroline nitrogen by the β-lactam-derived carboxylic acid of the hydrolysed product (Fig. 1). Therefore, the time-scale of the NMR analyses precludes their use for investigating the stereoselectivity of meropenem hydrolysis (and, likely, the hydrolysis of structurally related carbapenems).

It was predicted that the 1β-methyl group present in many carbapenems (R=CH_3_ in Fig. 1) may accelerate epimerisation in the carbapenem-derived products. In the (*R*)-Δ^1^ hydrolysis product epimer, the 1β-methyl group and the C-2 thioether substituent sterically clash, thereby favouring epimerisation to the more thermodynamically stable (*S*)-Δ^1^ product epimer. This is consistent with the greater levels of the (*S*)-Δ^1^ product epimers present at equilibrium, for both hydrolysis products and lactones [e.g., the ertapenem hydrolysis products exist as a ~5:1 ratio of the (*S*)-Δ^1^ and (*R*)-Δ^1^ epimers] (Supplementary Fig. S8). However, to date, the 1β-methyl group has been found to be required for the formation of carbapenem-derived lactones by class D SBLs^5^. Thus, we next examined the stereoselectivity of hydrolysis for imipenem, a carbapenem bearing a 1β-hydrogen (R=H in Fig. 1).

**Figure 3:**
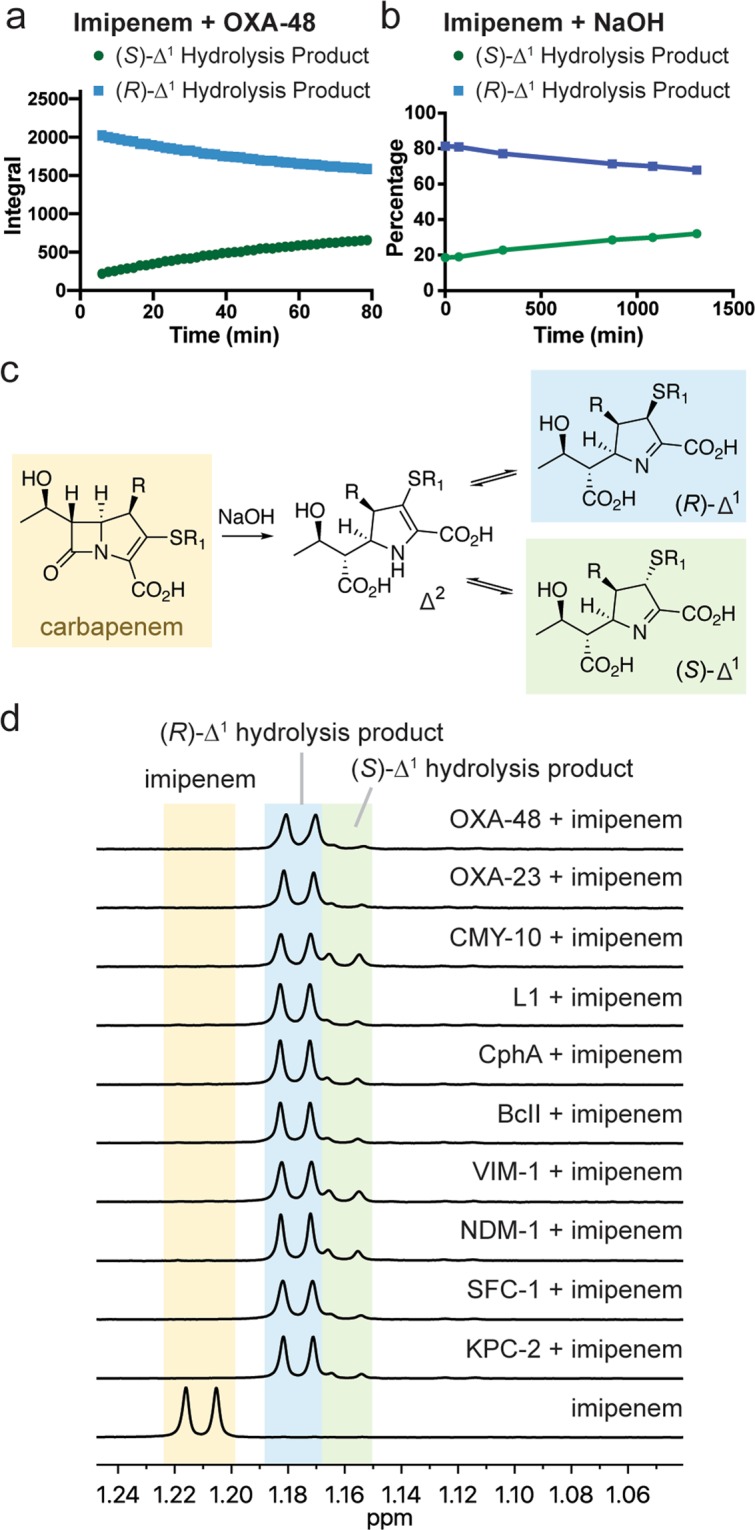
Stereoselectivity of enzymatic and non-enzymatic carbapenem hydrolysis. (**a**) NMR time course showing the levels of the (*R*)-Δ^1^ and (*S*)-Δ^1^ hydrolysis products formed by OXA-48 (5 µM) and imipenem (1 mM). Imipenem was fully hydrolysed by the first NMR measurement. (**b**) NMR time course showing the levels of the (*R*)-Δ^1^ and (*S*)-Δ^1^ hydrolysis products formed by treatment of imipenem (10 mM) with NaOH (100 mM). Imipenem was fully hydrolysed by the time of the first NMR measurement. (**c**) Scheme showing hydroxide-mediated degradation of a carbapenem, which is expected to initially form the Δ^2^ hydrolysis product. Rapid tautomerisation is then proposed to occur, with kinetically controlled protonation, forming the (*R*)-Δ^2^ hydrolysis product. Over time, tautomerisation and epimerisation occurs, forming the (*S*)-Δ^2^ hydrolysis product. (**d**) NMR spectra (600 MHz) showing the apparent preferential formation of the (*R*)-Δ^1^ hydrolysis product from imipenem (1 mM) by class A (KPC-2, SFC-1), class B (NDM-1, VIM-1, BcII, CphA, L1) class C (CMY-10), and class D (OXA-23, OXA-48) β-lactamases (5 µM, apart from CphA and L1, which were 1 µM and 0.15 µM, respectively). All spectra were acquired 5 min after mixing enzyme and imipenem, apart from CMY-10, which was acquired after 250 min. Note that the observations described in panel b suggest that the Δ^2^ imipenem hydrolysis product may be the nascent enzymatic product; this undergoes rapid tautomerisation giving the (*R*)-Δ^1^ hydrolysis product.

Following the hydrolysis of imipenem by OXA-48, a single product predominated at early time points; chemical shift assignments and NOESY NMR analyses led to its assignment as the (*R*)-Δ^1^ hydrolysis product epimer (Supplementary Table S7, Supplementary Fig. S9). Over time, the (*R*)-Δ^1^ hydrolysis product was observed to slowly interconvert to a second species (Fig. 3a), which was assigned as the (*S*)-Δ^1^ hydrolysis product epimer (Supplementary Table S7, Supplementary Fig. S10). This slow conversion suggests that the presence of a 1β-hydrogen does indeed slow the rate of non-enzymatic product epimerisation, compared to those products bearing a 1β-methyl group.

These observations may initially suggest that the class D SBLs stereoselectively degrade carbapenems to form the (*R*)-Δ^1^ lactone and (*R*)-Δ^1^ hydrolysis products. However, another scenario should be considered, i.e., in which the Δ^2^ product is enzymatically formed, and rapid kinetically controlled non-enzymatic tautomerisation occurs to produce the (*R*)-Δ^1^ epimer. Consistent with this proposal, non-enzymatic hydrolysis of carbapenems with sodium hydroxide is reported to (initially) give a non-equilibrium mixture of Δ^1^ epimers^13^. Using NMR, we examined the stereoselectivity of the non-enzymatic hydrolysis of meropenem (with a 1β-methyl group) and imipenem (with a 1β-hydrogen) by hydroxide. For both carbapenems, we observed the preferential initial formation of the (*R*)-Δ^1^ hydrolysis product epimer, which slowly epimerised to yield the (*S*)-Δ^1^ hydrolysis product (Fig. 3b,c, Supplementary Fig. S11). Thus, the apparent formation of the (*R*)-Δ^1^ product by a carbapenemase may be indistinguishable from the enzymatic formation of the Δ^2^ product, which can then rapidly tautomerise non-enzymatically to give the (*R*)-Δ^1^ product.

We next examined the stereoselectivity of the hydrolysis of imipenem (which lacks a 1β-methyl group) by carbapenemases belonging to other classes. Treatment of imipenem with the class A SBLs KPC-2 and SFC-1 and the class C SBL CMY-10 all resulted in the preferential initial formation of the (*R*)-Δ^1^ hydrolysis product (Fig. 3d). Similar selectivity was observed with MBLs belonging to subclasses B1 (NDM-1, BcII, VIM-1), B2 (CphA), and B3 (L1) (Fig. 3d). As indicated above, these spectra cannot distinguish whether the (*R*)-Δ^1^ product is the initially formed enzymatic product, or if, following the enzymatic formation of the Δ^2^ product, rapid tautomerisation occurs to form the (*R*)-Δ^1^ epimer.

## Discussion

**Figure 4:**
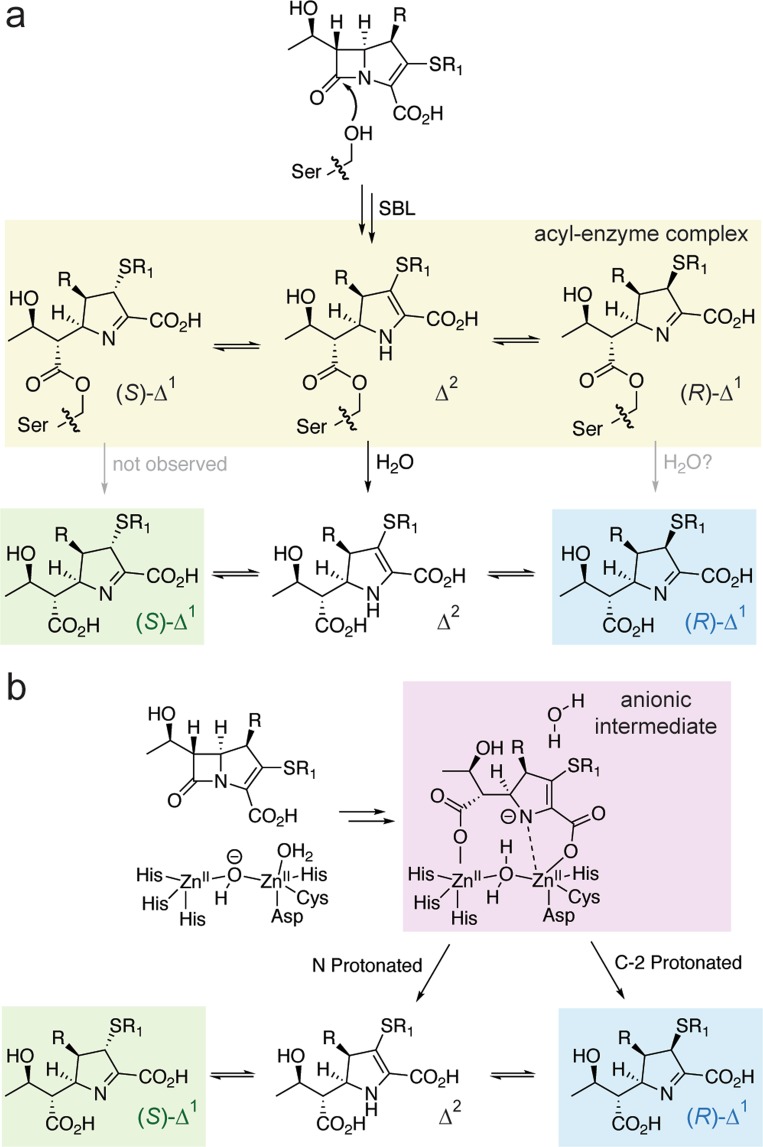
Mechanistic proposal for the stereoselectivity of carbapenem degradation by serine β-lactamases and metallo-β-lactamases. (**a**) Scheme showing the proposed outline mechanism of carbapenem degradation by SBLs. Based on our data and previous studies^12,14^, the Δ^2^ enamine form of the carbapenem-derived acyl-enzyme complex is likely hydrolysed more rapidly than the Δ^1^ forms. The Δ^2^ product, upon release from the enzyme, may then rapidly tautomerise to form the (*R*)-Δ^1^ product. Over time, the mixture undergoes further non-enzymatic epimerisation to reach equilibrium, yielding a mixture of the (*S*)-Δ^1^ and (*R*)-Δ^1^ products. Our NMR data, considered alone, cannot exclude the possibility that the (*R*)-Δ^1^ product is at least partially formed enzymatically (though we do not have direct evidence for this). Note that while lactone formation by class D SBLs is not represented in this scheme, its stereoselectivity is expected to be analogous (Supplementary Fig. S12). While the lactone product may then reacylate the enzyme, it is currently unclear which form(s) reacts with the enzyme most efficiently. (**b**) Scheme showing the proposed outline mechanism of carbapenem hydrolysis by a dinuclear MBL. Following the formation of an anionic intermediate^15^, protonation either occurs on the nitrogen (forming the Δ^2^ hydrolysis product) or on C-2 [forming the (*R*)-Δ^1^ hydrolysis product]. Note that our data indicates that NMR cannot differentiate between these two possibilities.

The apparent stereoselectivity, in which formation of the (*R*)-Δ^1^ and/or Δ^2^ products is favoured, has implications for understanding the mechanisms of carbapenem hydrolysis by both SBLs and MBLs. The three possible carbapenem-derived pyrroline ring forms (Fig. 1) have all been observed crystallographically in the acyl-enzyme complexes derived from carbapenems with different SBLs^7–11^. It is, however, unclear whether these structures represent catalytically viable complexes which are susceptible to hydrolysis, or if the observed form of the pyrroline ring reflects slow/inhibited degradation of the acyl-enzyme complex(es). Our observations suggest that hydrolysis (or lactonisation) of the carbapenem-derived acyl-enzyme complex occurs preferentially if the pyrroline ring is present in the Δ^2^ form [or, possibly, the (*R*)-Δ^1^ form] (Fig. 4a). These data are consistent with previous proposals that the Δ^2^ form of the pyrroline ring is required for efficient carbapenem hydrolysis by non-carbapenemases^13,14,19^. Thus, we propose that SBL carbapenemases efficiently degrade carbapenems in part by minimising the extent to which tautomerisation of the Δ^2^ pyrroline ring occurs in the acyl-enzyme complex, or potentially by facilitating tautomerisation of the Δ^1^ epimers back to the hydrolytically-susceptible Δ^2^ form.

During MBL-catalyzed carbapenem degradation, the anionic intermediate (in which the negatively charged pyrroline nitrogen is coordinated to an active site zinc)^15^ may be protonated either on the nitrogen, forming the Δ^2^ product, or on C-2, forming a Δ^1^ product epimer (Fig. 4b)^16^. Our observations with five diverse MBLs (Fig. 3d) suggest that their mechanisms of carbapenem degradation involve either protonation of the nitrogen to give the Δ^2^ product form, or protonation on C-2 to give the (*R*)-Δ^1^ product form (Fig. 4b). While a common mechanism for these five enzymes involving protonation on the nitrogen may be more likely, stereoselective protonation on C-2 could be preferred for currently undefined mechanistic reasons or as a consequence of mechanistic similarities between enzyme active sites. Following protonation, the hydrolysis product may then dissociate from the enzyme active site.

As highlighted by our study, care must be taken while investigating the role of the carbapenem-derived pyrroline ring in carbapenemase catalysis. Crystallographic studies require high-resolution diffraction data and careful modelling to properly identify the state of the pyrroline ring in the acyl-enzyme complexes derived from carbapenems and SBLs. These studies also require that the lifetime of the acyl-enzyme complex is sufficiently long so as to be observed in crystals. Thus, the extended times normally needed for crystallography may enable (non-catalytically relevant) tautomerisation of the pyrroline ring to occur^7^. Raman studies on carbapenemases also imply that the crystallographically observed structures are not necessarily representative of the solution state, particularly with respect to the form of the pyrroline ring^14^. The situation is further complicated with class D SBLs, as they can isomerise carbapenems to form β-lactones, which tautomerise/epimerise in solution and then may reacylate the enzyme^5^. Thus, at least with respect to the pyrroline ring, the crystallographically observed acyl-enzyme complexes of class D SBLs with carbapenems may not represent a species relevant to carbapenem degradation in solution.

NMR spectroscopic studies must also be interpreted carefully due to the non-enzymatic interconversion of the nascent enzyme-catalysed carbapenem degradation products^2^. Notably, our results contrast with the conclusions made in two recent reports in which NMR spectroscopy was used to investigate the stereoselectivity of carbapenem degradation by MBLs^16,17^. Feng *et al*. describe the exclusive formation of the (*S*)-Δ^1^ meropenem hydrolysis product by NDM-1^17^; however, as outlined above, the rapid non-enzymatic epimerisation of the hydrolysis products derived from carbapenems with 1β-methyl groups precludes the use of NMR to examine the stereoselectivity of hydrolysis. Lisa *et al*. also describe the preferential formation of the (*S*)-Δ^1^ hydrolysis product by the MBLs NDM-1, Sfh-I, and GOB-18 with imipenem^16^; however, their stereochemical assignments appear to be based on a study in which the stereochemistry of the imipenem hydrolysis products was not assigned^2^.

Following on from these studies, it is of interest to apply spectroscopic techniques to monitor the state of the carbapenem-derived pyrroline ring in the context of the covalent complexes derived from β-lactamases and transpeptidases. A promising approach has been applied to the acyl-enzyme complex derived from the l,d-transpeptidase from *Enterococcus faecium* with ertapenem, in which NMR experiments with selective filtering of the isotopically-labeled protein signals indicated that the carbapenem pyrroline ring is mostly present in the (*S*)-Δ^1^ form^20^. It is also of interest to further investigate the proposal that SBL carbapenemases manipulate the state of the pyrroline ring so as to favour the hydrolytically labile Δ^2^ form. This proposal raises the possibility of developing carbapenems which are resistant to carbapenemases (and which may be improved transpeptidase inhibitors) through modification of the steric and electronic properties of the carbapenem C-2 side chain, in a manner impacting on the tautomeric and epimeric state of the carbapenem pyrroline ring in the context of the acyl-enzyme complexes (with SBLs/transpeptidases) or metal-bound complex (with MBLs), in order to slow release from the active site.

## Methods

### NMR spectroscopy

NMR spectra were acquired using a Bruker AVIII HD 600 MHz spectrometer equipped with a BB-F/^1^H Prodigy N_2_ cryoprobe, a Bruker AVIII 700 MHz spectrometer equipped with a TCI cryoprobe, or a Bruker Avance 750 MHz spectrometer equipped with a TCI cryoprobe. All spectra were acquired at 298 K using 3 mm NMR tubes. Unless indicated otherwise, NMR samples were prepared in 50 mM sodium phosphate, pH 7.5, with 10% D_2_O. The water signal was suppressed using excitation sculpting or pre-saturation.

Chemical shift assignments were made based on ^1^H, COSY, TOCSY, HSQC, and HMBC spectra. ^1^H chemical shifts were referenced using an internal 3-(trimethylsilyl)-2,2,3,3-tetradeuteropropionic acid (TSP) standard, and ^13^C chemical shifts were referenced using the solvent lock signal. Spectra for the stereochemical assignment of the lactones derived from biapenem, doripenem, and meropenem employed a 1D SPFGSE 1 H,1H-NOESY pulse sequence with water pre-saturation and a 300 ms mixing time. Stereochemical assignments for the imipenem hydrolysis products were made using a 2D NOESY spectrum with zero-quantum suppression, excitation sculpting for water suppression, and a 600 ms mixing time.

Enzymatic carbapenem degradation was monitored using 1 mM of the indicated carbapenem and 5 µM of the indicated enzyme; zinc chloride (50 µM) was added to the metallo-β-lactamase reactions. Enzymes were produced and purified as described previously; purity (>95%) was confirmed by SDS-PAGE and mass spectrometric (MS) analyses^21–26^. Enzymes belonged to class A [*Klebsiella pneumoniae* carbapenemase-2 (KPC-2), *Serratia fonticola* carbapenemase-1 (SFC-1)] class B [New Delhi metallo-β-lactamase-1 (NDM-1), Verona integron-borne metallo-β-lactamase-1 (VIM-1), *Bacillus cereus* metallo-β-lactamase (BcII), carbapenemase hydrolysing *Aeromonas* (CphA), L1], class C [cephamycinase-10 (CMY-10)], and class D [oxacillinase-23 (OXA-23), OXA-48]. See the Supplementary Information for the methods used for the production and purification of CMY-10. Acquisition of the first NMR spectrum in the time course started 5 min after mixing the reaction components. Each spectrum in the time course consisted of 32 scans with a 1.7 s acquisition time, and a line broadening of 0.3 Hz was applied. The hydroxide-mediated carbapenem hydrolysis products were produced by treating 10 mM of the indicated carbapenem with 100 mM of sodium hydroxide, in 90% H_2_O, 10% D_2_O. The structures of these hydrolysis products were assigned based on ^1^H, COSY, TOCSY, HSQC, and NOESY spectra, acquired using a Bruker Avance III 700 MHz spectrometer equipped with a TXI Room Temperature probe at 273 K.

### Deuterium incorporation

The meropenem-derived β-lactone was produced by incubating meropenem with OXA-48 in 50 mM sodium phosphate, pH 7.5. The meropenem-derived hydrolysis product was produced by incubating meropenem with 5 µM NDM-1 in 50 mM sodium phosphate, pH 7.5, supplemented with 50 µM ZnCl_2_. The enzyme was removed from these mixtures using a 0.5 mL 10 kDa cut-off centrifugal filter (Amicon). A Sep-Pak light C18 cartridge (Waters) was used to purify the meropenem β-lactone. Following loading of the enzymatic reaction, the cartridge was washed with water to remove the hydrolysis product, and the meropenem-derived lactone was eluted using 25% acetonitrile in water, which was then lyophilised. The purity and concentration of the purified lactone and hydrolysis products were determined by ^1^H NMR (600 MHz), using TSP for quantification.

Deuterium incorporation was performed by preparing a 1 mM mixture of the meropenem-derived lactone and hydrolysis product in 18% H_2_O, 82% D_2_O. The sample was monitored using an NMR (600 MHz) time course of 60 consecutive ^1^H-NMR spectra, each consisting of 32 scans, with acquisition of the first spectrum beginning 5 minutes after mixing. Water suppression was accomplished by excitation sculpting with perfect echo. The extent of deuterium incorporation was determined based on the integration of the peak corresponding to the proton at the C-2 position of the pyrroline ring. The C-2 proton integrals were normalized according to the corresponding integrals obtained for a 1 mM sample of the meropenem-derived lactone and hydrolysis product prepared in 90% H_2_O, 10% D_2_O.

## Supplementary information

Supplementary Information

## Data Availability

All NMR data described in this study are available from the corresponding authors upon reasonable request.
