# Supplementary material for: Mechanistic Insights into β-Lactamase-Catalysed Carbapenem Degradation Through Product Characterisation
Source: Sci Rep. 2019 Sep 20;9:13608. doi: 10.1038/s41598-019-49264-0 (PMC6754457; doi:10.1038/s41598-019-49264-0)
Supplement: Supplementary file 1 — Supplementary Information [file 41598_2019_49264_MOESM1_ESM.pdf]

Supporting Information

Mechanistic Insights into β-Lactamase-Catalysed Carbapenem Degradation Through Product Characterisation

Christopher T. Lohans,[1,2*] Emily I. Freeman,[1] Emma van Groesen,[1] Catherine L. Tooke,[3] Philip Hinchliffe,[3] James Spencer,[3] Jürgen Brem,[1] and Christopher J. Schofield[1*]

[1]Department of Chemistry, University of Oxford, Oxford, UK, OX1 3TA.

[2]Department of Biomedical and Molecular Sciences, Queen's University, Kingston, ON, K7L 3N6, Canada.

[3]School of Cellular and Molecular Medicine, University of Bristol, Bristol, UK, BS8 1TD.

*Address correspondence to:

Prof. Christopher J. Schofield, Tel: +44 (0)1865 275625; Fax: +44 (0)1865 285002; Email: christopher.schofield@chem.ox.ac.uk

Dr. Christopher T. Lohans, Email: christopher.lohans@queensu.ca

Supporting Information Contents

Supplementary Methods	S3
Fig. S1. Structures of the carbapenems used in this study.	S5
NOESY spectra for stereochemical assignments	
Fig. S2. Meropenem-derived lactones	S6
Fig. S3. Doripenem-derived lactones	S7
Fig. S4. Biapenem-derived lactones	S8
Chemical shift assignments	
Table S1. Meropenem-derived lactones	S9
Table S2. Doripenem-derived lactones	S10
Table S3. Biapenem-derived lactones	S11
Fig. S5. Stereochemistry of lactones formed by OXA-23	S12
Fig. S6. Impact of pH on the OXA-48 ertapenem product distribution	S13
Chemical shift assignments	
Table S4. Meropenem hydrolysis products	S14
Table S5. Doripenem hydrolysis products	S15
Table S6. Biapenem hydrolysis products	S16
Fig. S7. HMBC signals assigned to Δ^2 carbapenem hydrolysis products	S17
Fig. S8. Ratios of carbapenem hydrolysis product stereoisomers with OXA-48	S18
Table S7. Chemical shift assignments for imipenem hydrolysis products	S19
Fig. S9. NOESY stereochemical analysis of the (*R*)-Δ^1 imipenem hydrolysis product	S20
Fig. S10. NOESY stereochemical analysis of the (*S*)-Δ^1 imipenem hydrolysis product	S21
Fig. S11. Stereochemical analysis of meropenem hydrolysis by hydroxide	S22
Fig. S12. Proposed outline pathways for carbapenem degradation by class D SBLs	S23
References	S24

Supplementary Methods

Production of CMY-10

The plasmid pNIC28-Bsa4-CMY-10 was prepared by ligation independent cloning (LIC),[1] using a codon-optimised synthetic gene as a template (GeneArt Gene Synthesis, Thermo Fisher Scientific). A starter culture of *Escherichia coli* BL21(DE3) (New England BioLabs) transformed with pNIC28-Bsa4-CMY-10 was grown overnight at 37°C, 180 rpm, in 2TY media supplemented with 50 μg/mL kanamycin (Apollo Scientific). This culture was used to inoculate (1 %) a larger 2TY culture, with 50 μg/mL kanamycin, which was grown at 37°C, 180 rpm until the culture reached an OD_{600} of 0.6. Isopropyl β-D-thiogalactopyranoside (IPTG) was then added to a final concentration of 0.5 mM, and the culture was incubated at 28 °C overnight. Cells were pelleted (6,500 × g, 10 min), frozen, and stored at -80°C.

The cell pellet was resuspended in HisTrap Buffer A (50 mM HEPES, pH 7.5, 20 mM imidazole, 500 mM NaCl) with added DNaseI, and the cells were lysed by sonication (SONIC Vibra-Cell). After pelleting the lysate (23,000 × g, 30 min), the lysate supernatant was passed through a 0.45 μm filter, and the filtrate was loaded onto a 5 mL HisTrap column (GE Life Sciences) pre-equilibrated with HisTrap Buffer A. After washing with 20 CV of HisTrap Buffer A, proteins were eluted using a gradient of 0-100% HisTrap Buffer B (50 mM HEPES, pH 7.5, 500 mM imidazole, 500 mM NaCl) over 8 CV, collecting 5 mL fractions. Fractions containing CMY-10 were pooled and concentrated using a 10 kDa molecular weight cut-off Amicon centrifugal filter (Merck Millipore). The concentrated CMY-10 was then loaded onto a 300 mL Superdex 200 column, pre-equilibrated with 50 mM sodium phosphate, pH 7.5. After eluting with 50 mM sodium phosphate, pH 7.5, fractions containing CMY-10 were pooled and concentrated as

described above, and frozen using liquid nitrogen. The identity and purity (>95%) of the purified protein was confirmed by mass spectrometry and SDS-PAGE.

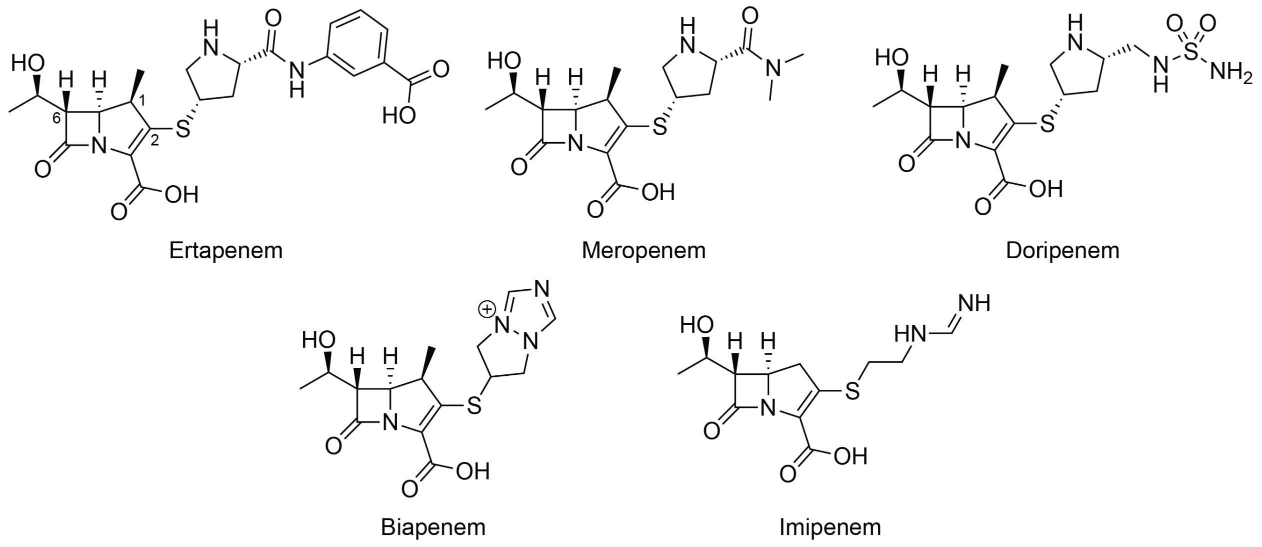

Figure S1. Structures of the carbapenems used in this study.

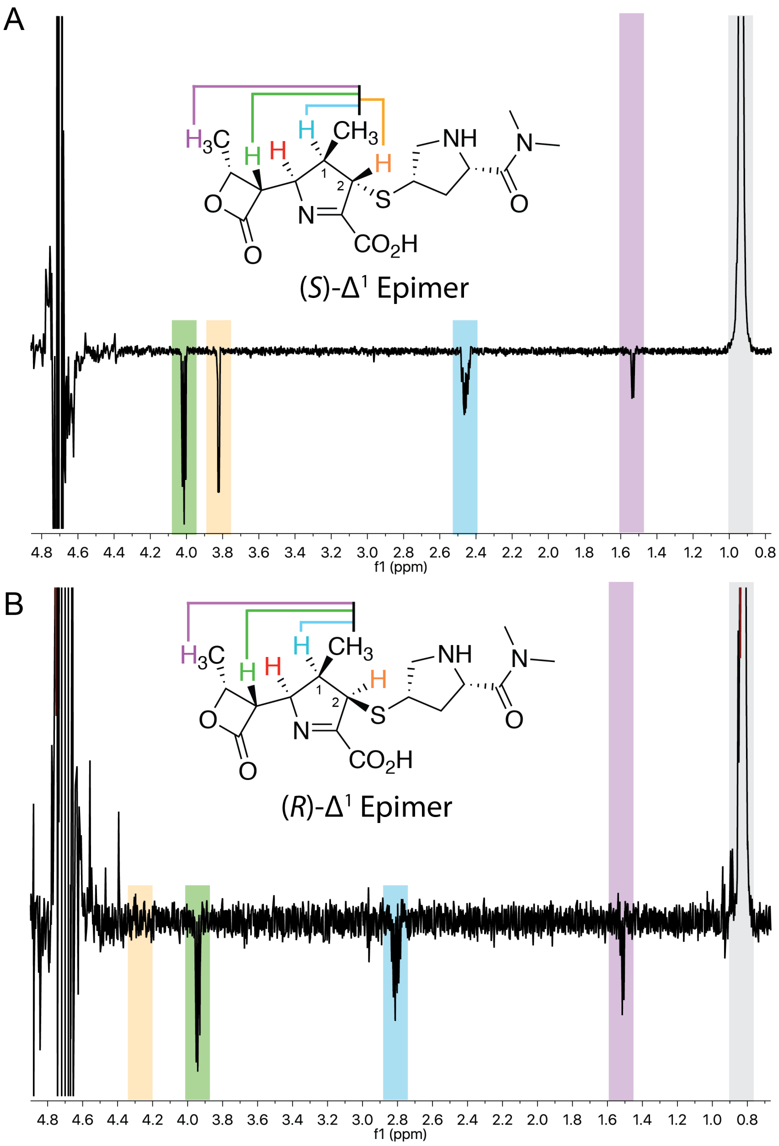

Figure S2. Stereochemical analysis of the meropenem-derived lactones with NOESY. 1D-nuclear Overhauser effect spectroscopy (NOESY) NMR spectra (700 MHz) of the (A) (*S*)-Δ^1 and (B) (*R*)-Δ^1 epimers of the meropenem-derived lactone, in which the 1β-methyl group was selectively irradiated. While the spectrum in panel A shows a strong correlation between the 1β-methyl group protons (black) and the proton on C-2 (orange), the corresponding signal is absent in the spectrum in panel B. These spectra and assignments are consistent with those previously reported for the ertapenem-derived lactones.[2] The nomenclature used for the different forms of the pyrroline ring are shown in Figure 1. The chemical shift assignments are listed in Table S1.

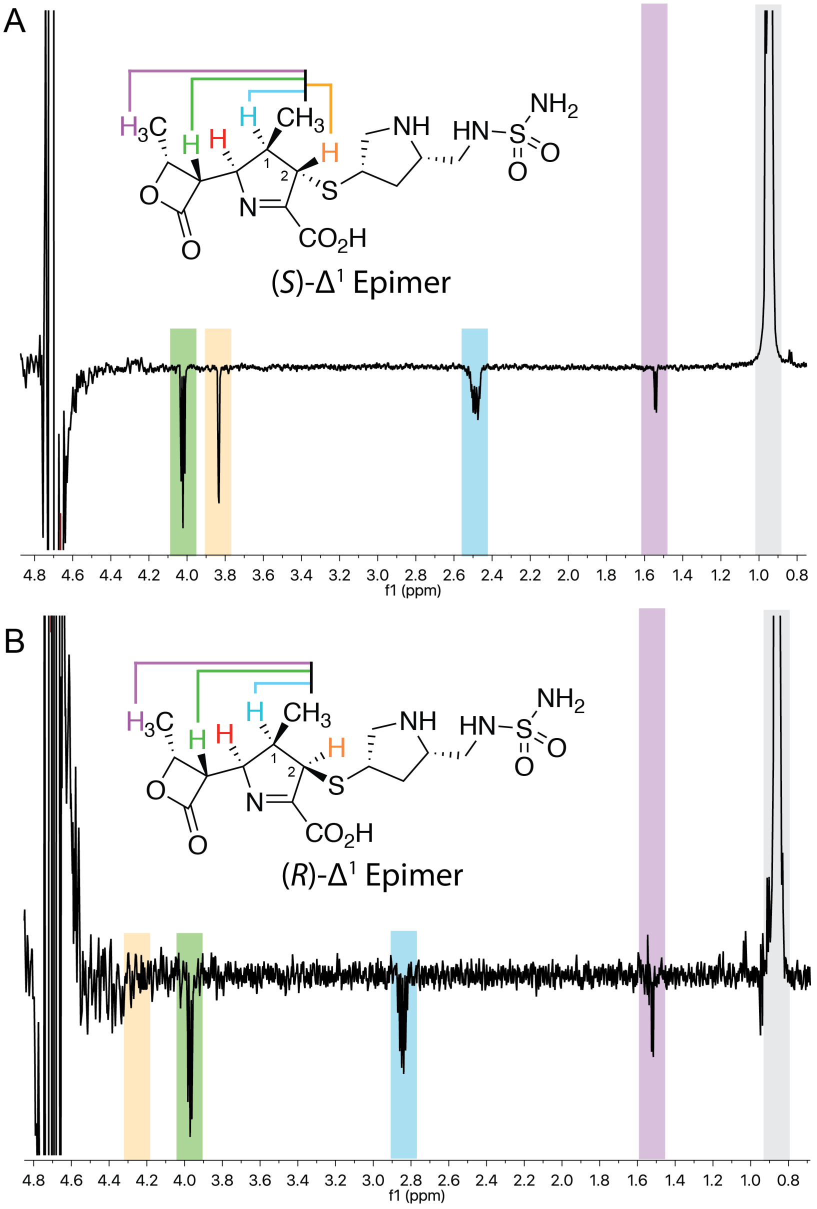

Figure S3. Stereochemical analysis of the doripenem-derived lactones with NOESY. 1D-NOESY NMR spectra (700 MHz) of the (A) (*S*)-Δ1 and (B) (*R*)-Δ1 epimers of the doripenem-derived lactone, in which the 1β-methyl group was selectively irradiated. While the spectrum in panel A shows a strong correlation between the 1β-methyl group protons (black) and the proton on C-2 (orange), the corresponding signal is absent in the spectrum in panel B. These spectra and assignments are consistent with those previously reported for the ertapenem-derived lactones.[2] The chemical shift assignments are listed in Table S2.

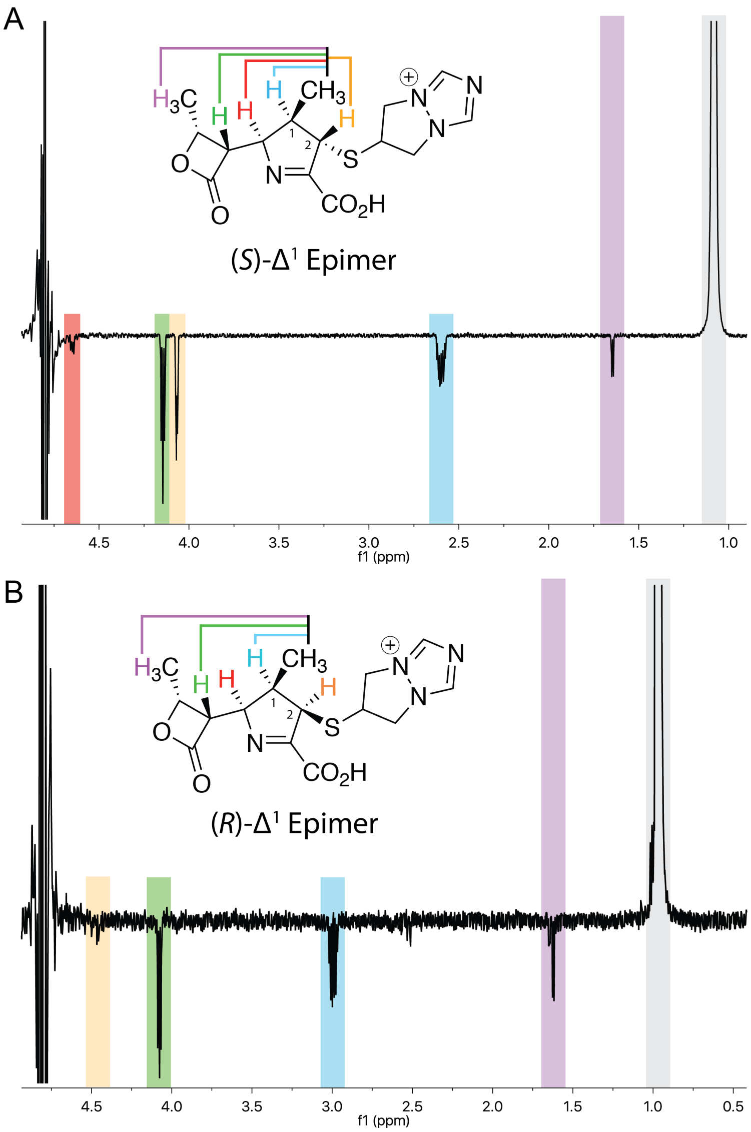

Figure S4. Stereochemical analysis of the biapenem-derived lactones with NOESY. 1D-NOESY NMR spectra (700 MHz) of the (A) (*S*)-Δ^1 and (B) (*R*)-Δ^1 epimers of the biapenem-derived lactone, in which the 1β-methyl group was selectively irradiated. While the spectrum in panel A shows a strong correlation between the 1β-methyl group protons (black) and the proton on C-2 (orange), the corresponding signal is absent in in the spectrum in panel B. These spectra and assignments are consistent with those previously reported for the ertapenem-derived lactones.[2] The chemical shift assignments are listed in Table S3.

Table S1. Chemical shift assignments for the meropenem-derived lactones.

	(*R*)-Δ[1] Epimer[a,b]		(*S*)-Δ[1] Epimer[b,c]	
**Position**	**^{13}C (ppm)**	**^{1}H (ppm)**	**^{13}C (ppm)**	**^{1}H (ppm)**
1		2.91	43.0	2.56
2		4.30	57.0	3.93
3			173.9	
5		4.42	69.0	4.63
6		4.04	52.6	4.12
7			172.8	
8		5.05	72.8	5.05
9		1.61	15.3	1.64
10				
11	10.6	0.92	13.9	1.04
12			51.8	3.34, 3.64
13			41.9	3.74
14			36.0	1.85, 2.93
15			57.6	4.60
16			169.5	
17, 18			36.0, 36.7	2.99, 3.08

[a]Full chemical shift assignments were not possible for the (*R*)-Δ[1] epimer of the meropenem-derived lactone due to spectral overlap with the (*S*)-Δ[1] epimer, which was present at higher levels.
[b]Stereochemical assignments based in part on the NOESY spectra shown in Figure S2.
[c]Chemical shift assignments from our previous report.[2]

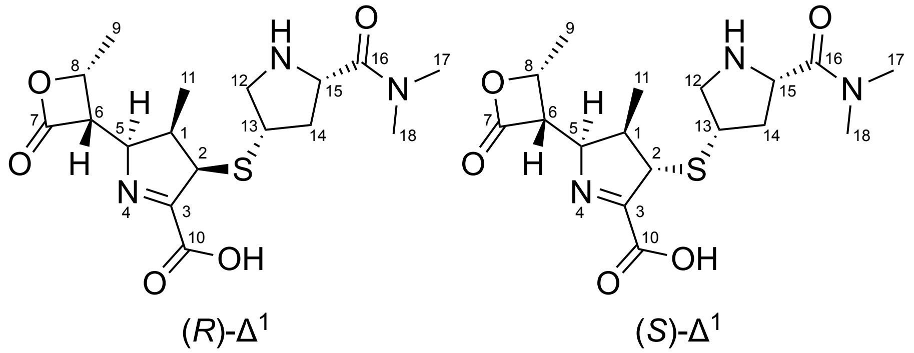

Table S2. Chemical shift assignments for the doripenem-derived lactones.

	(*R*)-Δ^1 Epimer[a,b]		(*S*)-Δ^1 Epimer[b,c]	
**Position**	**^{13}C (ppm)**	**^{1}H (ppm)**	**^{13}C (ppm)**	**^{1}H (ppm)**
1	38.4	2.87	43.1	2.52
2	56.0	4.25	57.2	3.86
3				
5		4.35	68.0	4.56
6	53.3	4.00	52.6	4.04
7			172.9	
8		4.98	73.4	4.99
9		1.54	15.3	1.57
10				
11	10.5	0.89	14.0	0.97
12			51.6	3.22, 3.54
13			40.7	3.66
14			35.3	1.63, 2.55
15			59.3	3.75
16			43.8	3.30, 3.38

[a]Full chemical shift assignments were not possible for the (*R*)-Δ^1 epimer of the doripenem-derived lactone due spectral overlap with the (*S*)-Δ^1 epimer, which was present at higher levels.
[b]Stereochemical assignments based in part on the NOESY spectra shown in Figure S3.
[c]Chemical shift assignments from our previous report.[2]

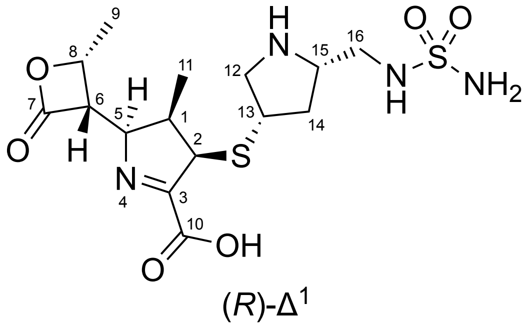

(*R*)-Δ^1

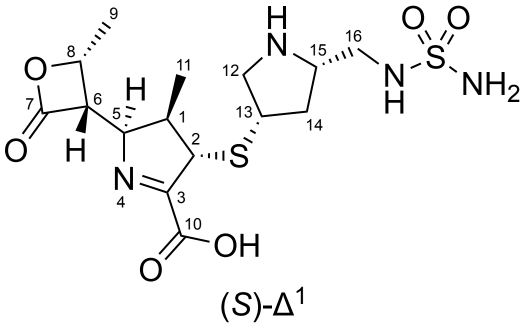

(*S*)-Δ^1

Table S3. Chemical shift assignments for the biapenem-derived lactones.

	(R)-Δ^1 Epimer[a,b]		Δ^2 Tautomer[a]		(S)-Δ^1 Epimer[b,c]	
**Position**	**^{13}C (ppm)**	**^{1}H (ppm)**	**^{13}C (ppm)**	**^{1}H (ppm)**	**^{13}C (ppm)**	**^{1}H (ppm)**
1	39.1	2.93		2.96	43.0	2.53
2	56.3	4.40	109.0		57.1	4.00
3					173.2	
5	68.0	4.39		4.15	68.0	4.58
6		4.01		4.07	52.5	4.08
7					172.8	
8		4.99		5.00	73.8	4.99
9		1.56		1.49	15.2	1.58
10						
11	10.6	0.90		0.94	13.7	1.02
12, 14					53.6	4.54, 5.06
13						
15, 16					143.4	8.96

[a]Full chemical shift assignments were not possible for the (R)-Δ^1 and Δ^2 forms of the biapenem-derived lactone due to spectral overlap with the (S)-Δ^1 form, which was present at higher levels.
[b]Stereochemical assignments based in part on the NOESY spectra shown in Figure S4.
[c]Chemical shift assignments from our previous report.[2]

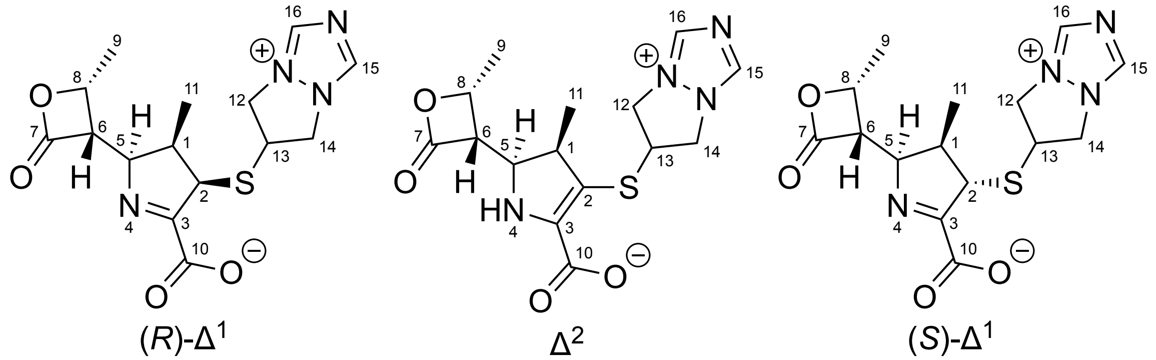

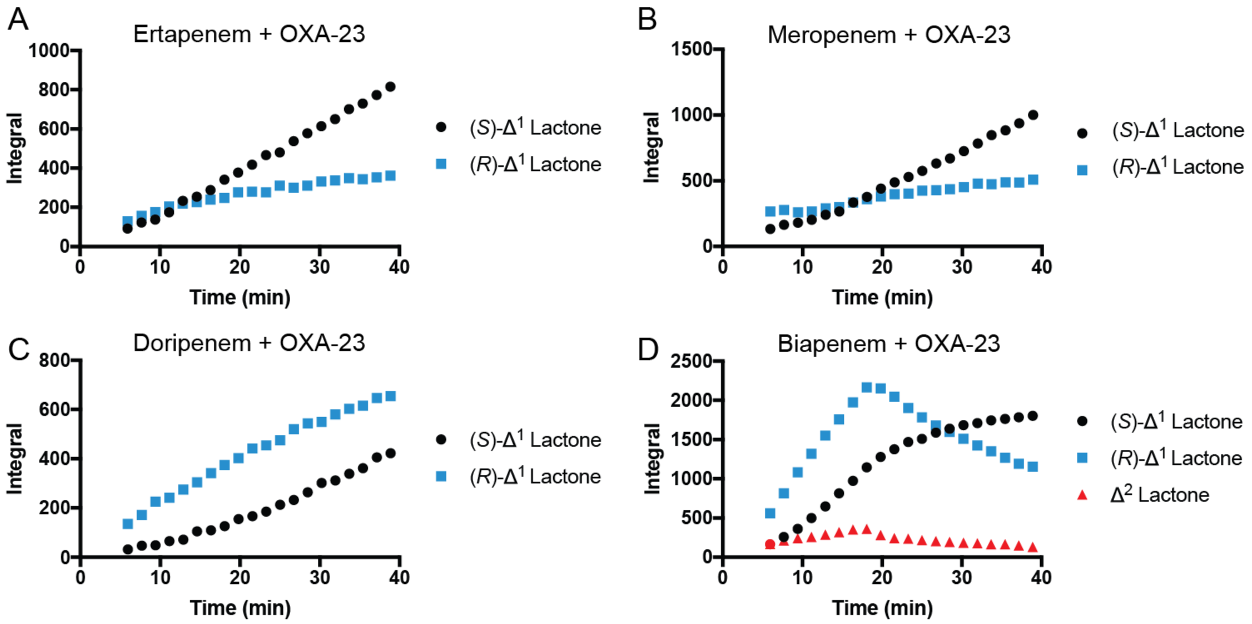

Figure S5. NMR time courses showing the stereochemistry of lactones formed by OXA-23 from carbapenems. Samples were made up of 5 μM OXA-23 and 1 mM (A) ertapenem, (B) meropenem, (C) doripenem, and (D) biapenem, and the extent of product formation was monitored by ^{1}H-NMR (600 MHz) spectroscopy. Integrals were measured for the resonances corresponding to the hydroxyethyl sidechain methyl group of the carbapenem-derived products.

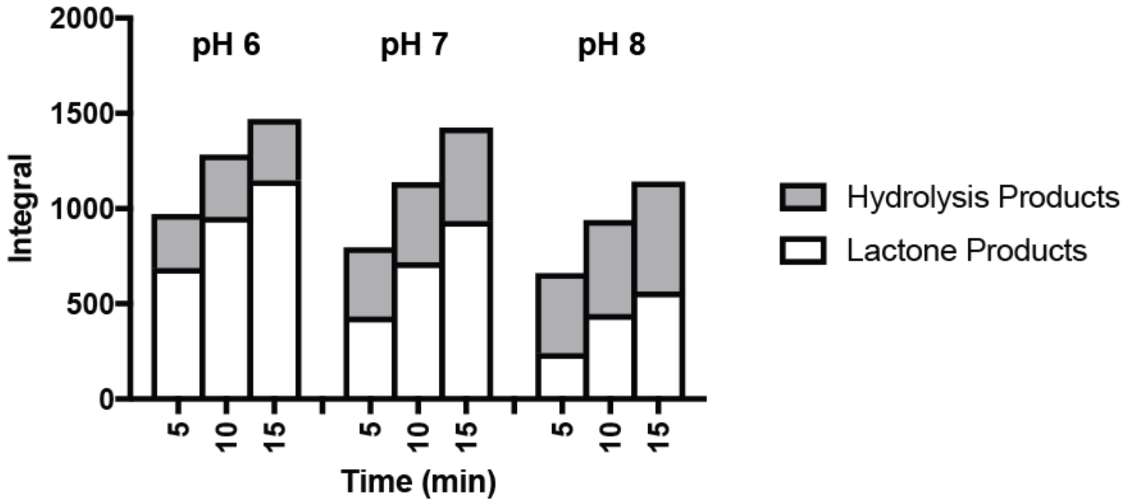

Figure S6. Impact of pH on the product distribution of OXA-48 with ertapenem. OXA-48 (5 μM) and ertapenem (1 mM) were prepared in 50 mM sodium phosphate, 10 % D_2O, at the indicated pH, and the products derived from ertapenem degradation were monitored by ^{1}H-NMR (600 MHz) spectroscopy. Integrals were measured for the resonances corresponding to the hydroxyethyl sidechain methyl group of the ertapenem-derived products; the values shown represent the combined integrals for the (*R*)-Δ^1 and (*S*)-Δ^1 epimeric forms of the hydrolysis and lactone products.

Table S4. Chemical shift assignments for the meropenem hydrolysis products.

	(*R*)-Δ[1] Epimer[a]		(*S*)-Δ[1] Epimer[b]	
**Position**	**^{13}C (ppm)**	**^{1}H (ppm)**	**^{13}C (ppm)**	**^{1}H (ppm)**
1	40.1	2.84	44.1	2.54
2	56.6	4.26	57.2	3.88
3	173.7		172.2	
5	72.8	3.98	73.0	4.32
6		2.63	56.0	2.61
7			180.1	
8			68.2	4.04
9		1.26	20.5	1.26
10				
11	10.1	0.89	13.8	1.06
12			52.0	3.35, 3.69
13			41.9	3.75
14			36.0	1.86, 2.94
15			57.6	4.65
16			169.2	
17, 18			35.9, 36.7	2.99, 3.08

[a]Full chemical shift assignments were not possible for the (*R*)-Δ[1] epimer of the meropenem hydrolysis product due to spectral overlap with the (*S*)-Δ[1] epimer, which was present at higher levels.

[b]Chemical shift assignments from our previous report.[2]

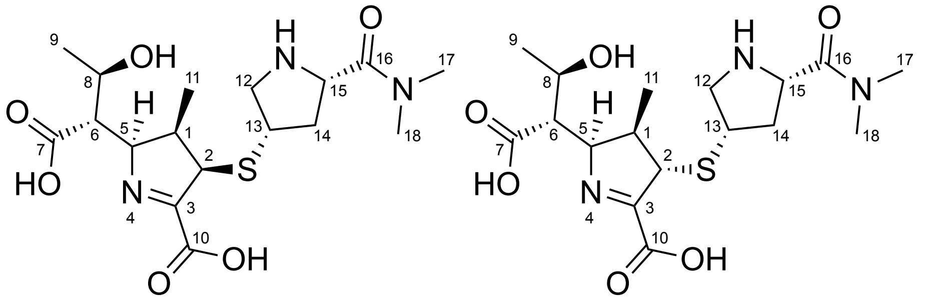

(*R*)-Δ[1]   (*S*)-Δ[1]

Table S5. Chemical shift assignments for the doripenem hydrolysis products.

	(*R*)-Δ[1] Epimer[a]		(*S*)-Δ[1] Epimer[b]	
**Position**	**^{13}C (ppm)**	**^{1}H (ppm)**	**^{13}C (ppm)**	**^{1}H (ppm)**
1	39.9	2.88	43.9	2.55
2	56.4	4.28	57.1	3.88
3			172.4	
5	72.7	3.99	73.0	4.33
6		2.63	55.9	2.61
7			180.0	
8			68.2	4.05
9		1.26	20.5	1.26
10				
11	10.1	0.94	13.8	1.06
12			51.4	3.32, 3.74
13			40.3	3.75
14			35.0	1.73, 2.65
15			59.2	3.86
16			43.4	3.39, 3.48

[a]Full chemical shift assignments were not possible for the (*R*)-Δ[1] epimer of the doripenem hydrolysis product due to spectral overlap with the (*S*)-Δ[1] epimer, which was present at higher levels.

[b]Chemical shift assignments from our previous report.[2]

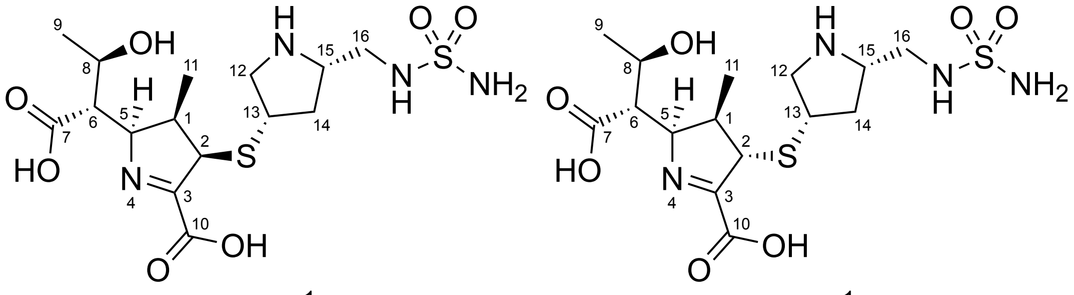

(*R*)-Δ[1] (*S*)-Δ[1]

Table S6. Chemical shifts for the biapenem hydrolysis products.

	**(*R*)-Δ[1] Epimer[a]**		**(*S*)-Δ[1] Epimer[b]**	
**Position**	**^{13}C (ppm)**	**^{1}H (ppm)**	**^{13}C (ppm)**	**^{1}H (ppm)**
1	39.9	2.94	43.9	2.55
2		4.40	57.3	4.00
3				
5		4.08	72.8	4.37
6		2.62	56.1	2.61
7			180.2	
8			68.0	4.04
9		1.27	20.6	1.26
10				
11	10.2	0.97	13.6	1.10
12, 14			53.6	4.61, 5.13
13				
15, 16			143.3	9.02

[a]Full chemical shift assignments were not possible for the (*R*)-Δ[1] and Δ[2] forms of the biapenem hydrolysis product due to spectral overlap with the (*S*)-Δ[1] form, which was present at higher levels. [b]Chemical shift assignments from our previous report.[2]

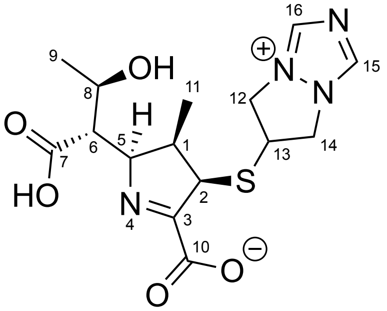

(*R*)-Δ[1]

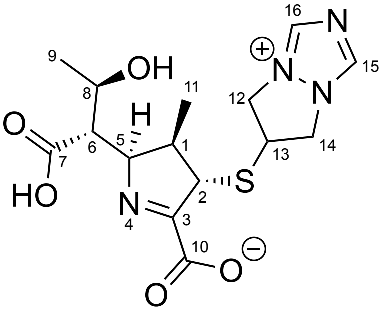

(*S*)-Δ[1]

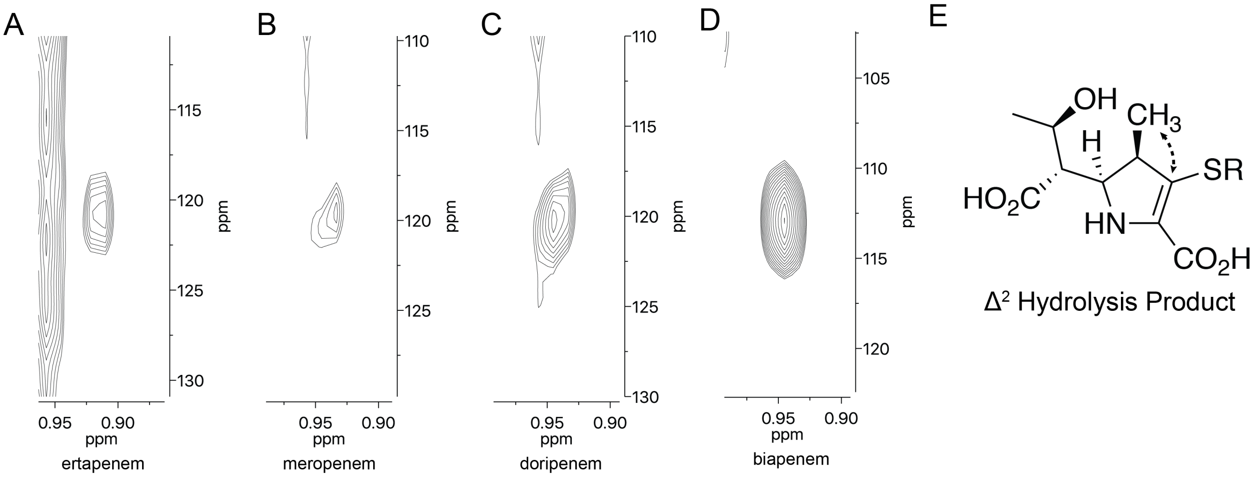

Figure S7. HMBC signals assigned to the Δ^2 carbapenem hydrolysis products. Views from HMBC spectra (700 MHz) of the hydrolysis products derived from: (A) ertapenem, (B) meropenem, (C) doripenem, and (D) biapenem. (E) Chemical structure of the Δ^2 form of a carbapenem-derived hydrolysis product. The signals shown in panels A-D are assigned as resulting from correlation between the protons on the 1β-methyl group (~0.90-0.95 ppm) and C-2 (~110-125 ppm) in the Δ^2 product (represented by the arrow on the structure). Note that the ^{13}C chemical shift of C-2 is consistent with a carbon-carbon double bond between C-2 and C-3, while the ^{13}C chemical shift of C-2 in the corresponding (*S*)-Δ^1 hydrolysis products appears at ~57 ppm. Due to the low levels of the Δ^2 hydrolysis product present, and spectral overlap with the other tautomeric forms of the hydrolysis product(s), further chemical shift assignments were not possible.

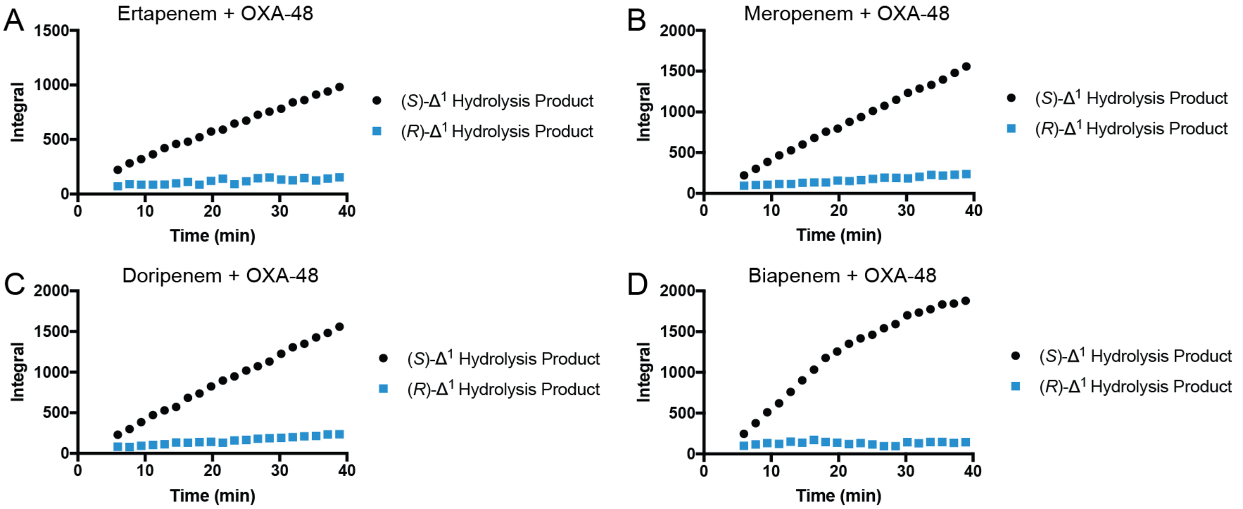

Figure S8. Ratios of carbapenem hydrolysis product stereoisomers with OXA-48 from carbapenems. Time course showing the integrals of the (*S*)-Δ^1 and (*R*)-Δ^1 hydrolysis products formed by OXA-48 (5 µM) with 1 mM of (A) ertapenem, (B) meropenem, (C) doripenem, and (D) biapenem. Samples were prepared in 50 mM sodium phosphate, pH 7.5, with 10 % D_2O, and the extent of product formation was monitored by NMR spectroscopy. Integrals were measured for the resonances corresponding to the hydroxyethyl sidechain methyl group and/or 1β-methyl group of the carbapenem-derived products.

Table S7. Chemical shift assignments for the imipenem hydrolysis products.

	**(*R*)-Δ^1 Form[a,b]**		**(*S*)-Δ^1 Form[b,c]**	
**Position**	**^{13}C (ppm)**	**^{1}H (ppm)**	**^{13}C (ppm)**	**^{1}H (ppm)**
1	34.8	1.68, 2.69	36.3	2.15, 2.35
2	49.8	4.13	49.7	4.18
3	173.2			
5	70.0	4.24	71.2	4.42
6	61.7	2.35	61.4	2.32
7	179.6		179.7	
8	67.5	4.12	67.5	4.06
9	21.2	1.19	21.0	1.17
10				
11	27.8	2.80	28.4	2.83
12	41.0	3.47	36.7	3.52
13	154.5	7.74	157.7	7.79

[a]Chemical shift assignments from our previous report.[2] Note that the stereochemistry of this hydrolysis product was not investigated in this previous work.

[b]Two sets of signals were observed, likely corresponding to geometric isomerism in the *N*-formimidoyl sidechain.[3]

[c]Stereochemical assignments based in part on the NOESY spectra shown in Figure S9 and S10.

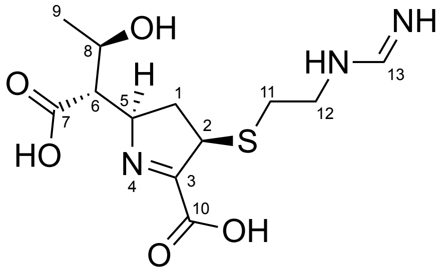

(*R*)-Δ^1

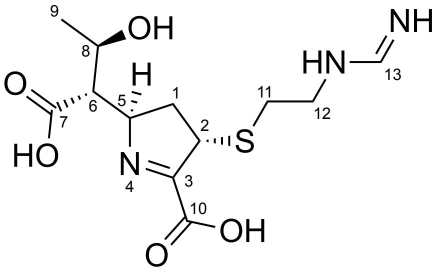

(*S*)-Δ^1

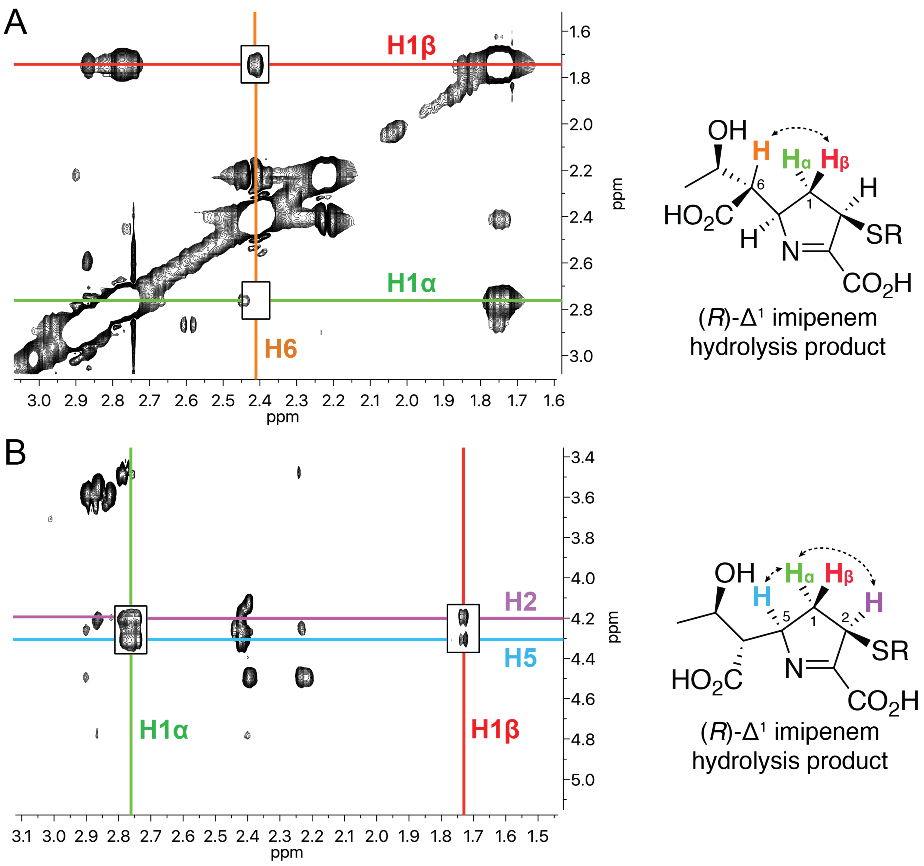

Figure S9. Stereochemical analysis of the (*R*)-Δ[1] imipenem-derived hydrolysis product by NOESY. Selected views from a 2D-NOESY spectrum (700 MHz) of the hydrolysis products derived from imipenem. (A) A correlation was observed between the proton on C-6 (2.41 ppm; orange) and one of the diastereotopic C-1 protons (1.74 ppm; red), while no corresponding cross-peak was observed for the other C-1 proton (2.77 ppm; green). Therefore, the proton at 1.74 ppm (red) was assigned as being in the *pro*-R position (β), and the proton at 2.77 ppm (green) was assigned as being in the *pro*-S position (α). (B) Strong correlations are present between the *pro*-S proton (α; green) and the protons on C-2 (purple) and C-5 (blue), while weak correlations occur between the *pro*-R proton (β; red) and the C-2 (purple) and C-5 (blue) protons. These observations suggest that the C-2 and C-5 protons both have a cis orientation relative to the *pro*-R proton, thus indicating that this is the (*R*)-Δ[1] form of the imipenem hydrolysis product. Note that the chemical shifts observed in this spectrum deviated (approx. 0.06 ppm) from other experiments, likely due to the greater concentration of imipenem used. The observed NOEs are represented by arrows on the chemical structure. The chemical shift assignments are listed in Table S7.

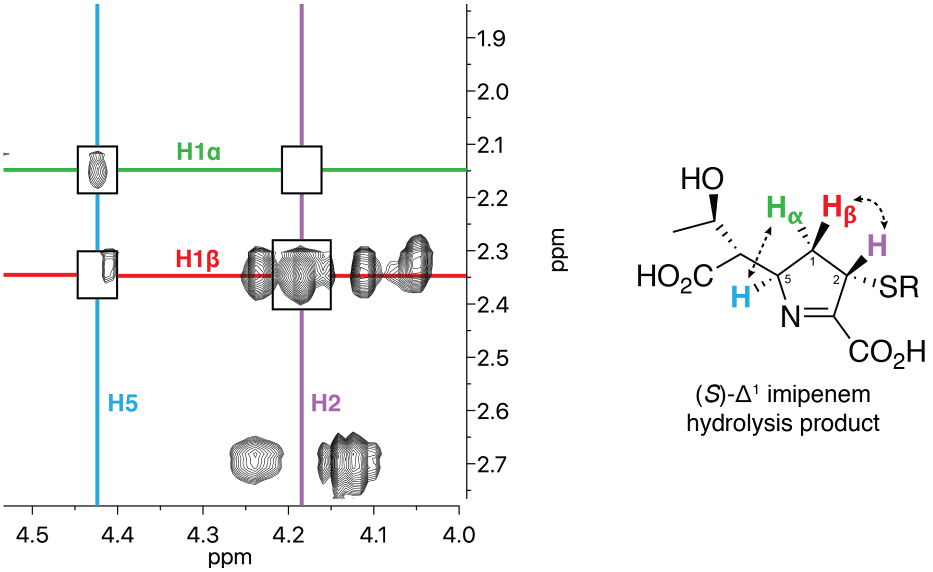

Figure S10. Stereochemical analysis of the (*S*)-Δ^1 imipenem-derived hydrolysis product by NOESY. (A) View from a 2D-NOESY spectrum (700 MHz) of the hydrolysis products derived from imipenem. A correlation was observed between the proton on C-5 (4.42 ppm; blue) and one of the diastereotopic C-1 protons (2.15 ppm; green), while no corresponding cross-peak was observed for the other C-1 proton (2.35 ppm; red). Therefore, the proton at 2.15 ppm (green) was assigned as being in the *pro*-S position (α), and the proton at 2.35 ppm (red) was assigned as being in the *pro*-R position (β). Another correlation was observed between the proton on C-2 (4.18 ppm; purple) and one of the diastereotopic C-1 protons (2.35 ppm; red), while no corresponding cross-peak was observed for the other C-1 proton (2.15 ppm; green). Based on these stereochemical assignments, the pyrroline ring was assigned as being in the (*S*)-Δ^1 form. The observed NOEs are represented by arrows on the structure. The chemical shift assignments are listed in Table S7.

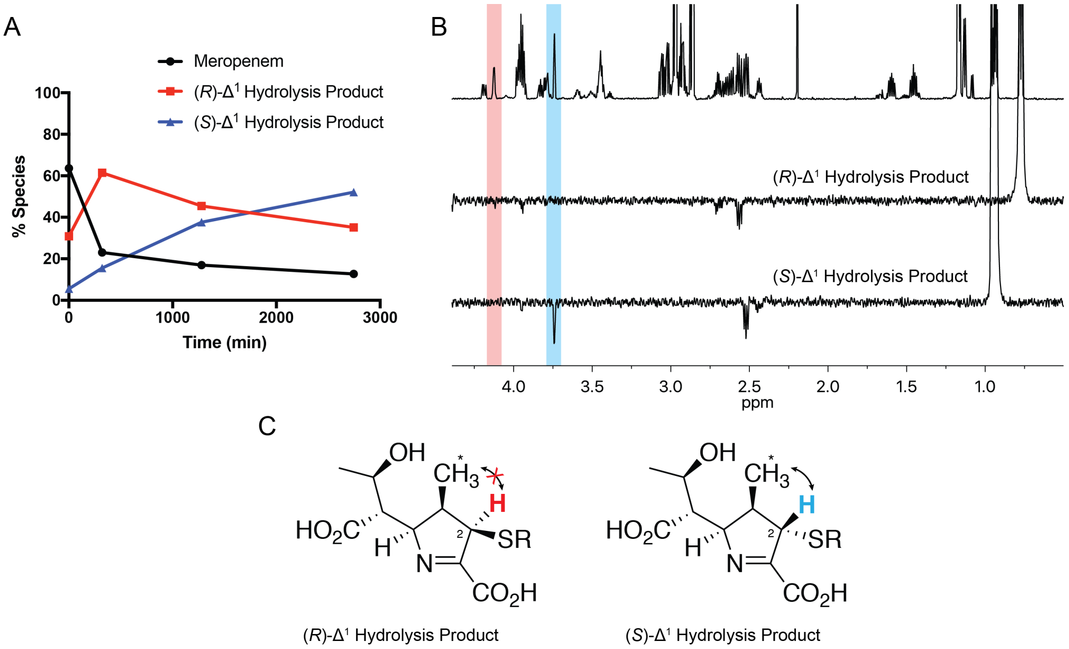

Figure S11. Stereochemical analysis of meropenem hydrolysis by hydroxide. (A) NMR time course for the degradation of meropenem (10 mM) by sodium hydroxide (100 mM), showing the relative levels of intact meropenem, and the (*R*)-Δ^1 and (*S*)-Δ^1 forms of the meropenem hydrolysis product. The percentages shown were determined based on the integrals obtained for the 1β-methyl groups of the corresponding species. (B) ^{1}H-NMR spectrum (700 MHz) of the meropenem hydroxide mixture (after 21 h), and 1D SPFGSE 1H,1H-NOESY spectra irradiating the 1β-methyl group of the (*R*)-Δ^1 and (*S*)-Δ^1 hydrolysis products (indicated with asterisks in panel C). (C) Scheme explaining the C-2 stereochemical assignments made in panel B on the basis of NOESY spectra. Irradiation of the 1β-methyl group in the (*S*)-Δ^1 hydrolysis product gives rise to a signal consistent with the C-2 hydrogen (highlighted in blue in panel B, shown in blue in panel C), while irradiation of the 1β-methyl group in the (*R*)-Δ^1 hydrolysis product does not give rise to a similar enhancement to the C-2 hydrogen (highlighted in red in panel B, shown in red in panel C).

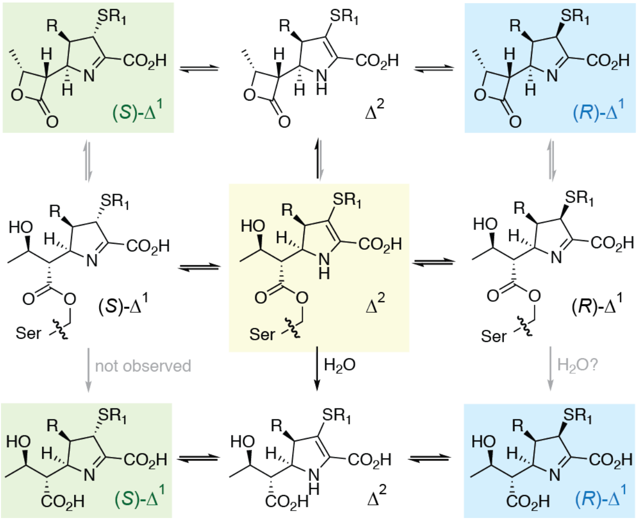

Figure S12. Proposed outline pathways for carbapenem degradation by class D SBLs. As in Figure 4A, the evidence implies that the Δ^2 enamine form of the carbapenem-derived acyl-enzyme complex (highlighted in yellow) likely undergoes hydrolysis and lactone formation more rapidly than the Δ^1 imine forms. Upon release from the class D SBL, the Δ^2 hydrolysis and lactone products may then rapidly tautomerise to the (*R*)-Δ^1 products (highlighted in blue). Over time, the mixture undergoes non-enzymatic epimerization, yielding a mixture of the (*S*)-Δ^1 and (*R*)-Δ^1 products (highlighted in green and blue, respectively). Note that our observations made through NMR, considered alone, do not exclude the possibility that the (*R*)-Δ^1 product is at least partially formed enzymatically during catalyses (though we do not have direct evidence for this). Note also that both Δ^1 and Δ^2 forms may manifest during carbapenem-mediated inhibition of SBLs and PBPs, though our results suggest the former may be preferred. Although carbapenem-derived lactones can reacylate class D SBLs,[2] it is not yet known how the structure of the pyrroline ring influences this activity.

References

1. Gileadi, O. et al. High throughput production of recombinant human proteins for crystallography. *Methods Mol Biol* **426**, 221-246 (2008).
2. Lohans, C. T. et al. A New Mechanism for β-Lactamases: Class D Enzymes Degrade 1β-Methyl Carbapenems through Lactone Formation. *Angew. Chem. Int. Ed. Engl.* **57**, 1282-1285 (2018).
3. Ratcliffe, R. W. et al. Studies on the structures of imipenem, dehydropeptidase I-hydrolyzed imipenem, and related analogs. *J. Org. Chem.* **54**, 653-660 (1989).
